# Transcriptome sequencing and SNP detection in *Phoebe chekiangensis*

**DOI:** 10.7717/peerj.3193

**Published:** 2017-05-10

**Authors:** Bing He, Yingang Li, Zhouxian Ni, Li-an Xu

**Affiliations:** 1 Co-Innovation Center for Sustainable Forestry in Southern China, Nanjing Forestry University, Nanjing, China; 2 Zhejiang Academy of Forestry, Zhejiang Academy of Forestry, Hangzhou, China

**Keywords:** *Phoebe chekiangensis*, SNP prediction, Next-generation sequencing, Sanger method, Software comparison

## Abstract

**Background:**

*Phoebe chekiangensis* is a rare tree species that is only distributed in south-eastern China. Although this species is famous for its excellent wood properties, it has not been extensively studied at the molecular level.

**Results:**

Here, the transcriptome of *P. chekiangensis* was sequenced using next-generation sequencing technology, and 75,647 transcripts with 48,011 unigenes were assembled and annotated. In addition, 162,938 putative single nucleotide polymorphisms (SNPs) were predicted and 25 were further validated using the Sanger method.

**Conclusion:**

The currently available SNP prediction software packages showed low levels of correspondence when compared. The transcriptome and SNPs will contribute to the exploration of *P. chekiangensis* genetic resources and the understanding of its molecular mechanisms.

## Introduction

*Phoebe chekiangensis*, which belongs to Lauraceae, is a tree species with a high economic value worldwide that is mainly distributed in south-eastern China. *P. chekiangensis* is the major source of the well-known wood ‘Golden Phoebe’. This wood has a superb reputation for its high-quality properties, such as its strong resistance to decomposition and dense texture (Gao et al., 2016). In addition to being widely used as timber or furniture in the imperial palace over the centuries, *P. chekiangensis* is a suitable garden plant species because of its outstanding tree morphology. However, due to its narrow distribution and slow growth, limited research has been conducted on this species, including studies of its general genomic studies.

Single nucleotide polymorphisms (SNPs) are widely used as genetic markers in association studies to understand inter-individual differences because of their characteristics of high frequency and binary variation patterns (Collins, Brooks & Chakravarti, 1998). Compared with traditional technologies, next-generation sequencing (NGS) technologies are usually more suitable for SNP identification because of their high throughput, although many artifacts are caused by systemic or random error. Researches on SNP identification and association studies have been carried on in many species (Martin et al., 2008; Ratan et al., 2015); however, very few SNPs are available in tree species because of the limited transcriptomic and genomic resources. Additionally, more validation work on putative SNP predicted by software using molecular experimental methods are required.

Over the past few years, NGS technology has led to profound changes in genomic and genetic research, with faster sequencing rates and continually decreasing costs (Mardis, 2008). Among the currently available NGS sequencing platforms, Illumina Hiseq2000 is relatively more cost effective, and it has been widely applied in the deep sequencing of model and non-model species (De et al., 2013). Because the determination of expressed sequence tags (ESTs) is an effective method for understanding the molecular mechanisms underlying physiological and morphological traits, for the first time, we sequenced the transcriptome of *P. chekiangensis* using Illumina HiSeqTM 2000 platform. This will help better understand and protect this rare tree species, and may aid in revealing the genetic principles of *P. chekiangensis*.

## Materials and Methods

### Sample collection and preparation

Leaves from a mature *P. chekiangensis* tree were collected in Zhejiang Academy of Forestry. Then the leaves were quickly frozen in liquid nitrogen and stored at −80 °C until RNA extraction. RNA degradation and contamination was monitored on 1% agarose gels. RNA integrity was assessed using the RNA Nano 6000 Assay Kit of the Agilent Bioanalyzer 2100 system (Agilent Technologies, Palo Alto, CA, USA). RNA purity was determined using the NanoPhotometer^®^ spectrophotometer (IMPLEN, Westlake Village, CA, USA). In addition, RNA concentration were measured using a Qubit^®^ RNA Assay Kit in a Qubit^®^ 2.0 Fluorometer (Life Technologies, Carlsbad, CA, USA).

### Library preparation for transcriptome sequencing

In each sample, 3 µg RNA was used as input for the RNA sample preparations. NEBNext^®^ Ultra™ RNA Library Prep Kit for Illumina^®^ (New England Biolabs (NEB), Beverly, MA, USA) was used to generate the sequencing libraries following the manufacturers’ recommendations. Briefly, with using poly-T oligo-attached magnetic beads, mRNA was purified from total RNA. Fragmentation was then performed under elevated temperatures in NEB Next First Strand Synthesis Reaction Buffer (5×). First strand cDNA was synthesized using a random hexamer primer and M-MuLV Reverse Transcriptase (RNase H-), and second strand cDNA synthesis was subsequently performed using DNA Polymerase I and RNase H. Using exonuclease/polymerase activities, remaining overhangs were converted into blunt ends. NEB Next Adaptor with hairpin loop structures were ligated to prepare for hybridization after the adenylation of 3′ ends of DNA fragments. The library fragments were purified with AMPure XP system (Beckman Coulter, Beverly, MA, USA) in order to select cDNA fragments ranging from 150 bp to 200 bp. Afterwards, 3 µl USER Enzyme was used with size-selected, adaptor-ligated cDNA at 37 °C for 15 min followed by 5 min at 95 °C before PCR. The library quality was assessed on the Agilent Bioanalyzer 2100 system and PCR reaction was performed with Phusion High-Fidelity DNA polymerase, Universal PCR primers and Index (X) Primer. At last, PCR products were purified (AMPure XP system).

### Clustering and sequencing

According to the manufacturers’ instructions, the clustering of the index-coded samples was performed on a cBot Cluster Generation System using TruSeq PE Cluster Kit v3-cBot-HS (Illumina). After cluster generation, the library preparations were sequenced on an Illumina Hiseq 2000 platform and paired-end reads were generated.

The raw data of fastq format was firstly processed through in-house perl scripts (File S4). After removing reads containing adapters (reads containing more than 5 adapter-polluted bases were regarded as adaptor-polluted reads and would be filtered out), reads containing poly-Ns accounting for more than 5% and low quality reads (reads with the number of low quality bases (phred quality <19) accounting for more than 15% of the total bases) from the raw data, clean data (clean reads) were subsequently obtained. At the same time, Q20, Q30, GC-content and sequence duplication levels of the clean data were calculated. All of the downstream analyses were based on clean data of high quality.

Before transcriptome assembly, we counted the clean reads number for each transcript form 5′ end to 3′ end, and obtained the reads distribution for overall transcripts. Transcriptome assembly was accomplished based on the left.fq and right.fq using Trinity (v2012-10-5) (Haas et al., 2013) with min_kmer_cov set to 2, and all other parameters were set as default accordingly.

### Functional annotation of unigenes and quantification of gene expression levels

After assembly, the longest transcript was defined accordingly as a unigene. Then, the unigenes were annotated based on the following databases: NCBI non-redundant protein sequences; NCBI non-redundant nucleotide sequences; Pfam; Clusters of Orthologous Groups of proteins; KEGG Ortholog and GO. The coding sequences and amino acids were determined based on standard codon usage table. Unigenes which couldn’t be blasted to neither database were processed by ESTScan (Iseli, Jongeneel & Bucher, 1999).

Gene expression levels were estimated by RSEM (Li & Dewey, 2011) for each sample: 1. Clean data were mapped back onto the assembled transcriptome; 2. Readcount for each gene was obtained from the mapping results.

### SNP calling and SSR prediction

SNP prediction was performed using the following workflow: the clean reads were firstly aligned with the transcripts that were assembled by Trinity, and then the duplicated reads and multi-mapped reads were filtered. Subsequently, the alignment results were sorted according to the transcripts’ locations. SOAPsnp (v1.03) was used for SNP calling based on the sorted data, and initial raw prediction results were obtained (Li et al., 2009b). After further filtering based on their quality values, sequencing depths and SNP separation distances, final SNP prediction results were acquired.

SSRs of the transcriptome were identified using MISA (http://pgrc.ipk-gatersleben.de/misa/misa.html), and primers for each SSR were designed using Primer3 (http://primer3.sourceforge.net/releases.php).

### SNP validation

Leaves of 114 samples including 9 populations were first collected during November and December in 2011–2012 (Table 1). PCR reactions were performed using the following procedure: an initial denaturation for 5 min at 94 °C, 30 cycles of 30 s at 94 °C, 30 s at the locus-specific annealing temperature, and 40 s at 72 °C, followed by a final extension of 1 min at 72 °C. A typical 10 µl reaction contained 1 × buffer, 2.5 mM MgCl_2_, 0.2 mM of each dNTPs, 0.25 µM of each primer, 0.25 U of Taq DNA polymerase (Takara, Kusatsu, Shiga, Japan) and 25 ng genomic DNA.

**Table 1:** Geographical locations and main climatic conditions for nine populations of *P. chekiangensis*.

Sampling site	Population type	No. of individuals sampled	Longitude (E)	Latitude (N)	Altitude (m)
Xihu Lake, Hangzhou	Wild population	30	120.06°	30.12°	135
Yinzhou, Ningbo	Wild population	8	121.47°	29.47°	280
Lin’an	Population of ancient trees	7	119.26°	30.19°	355
Taishun	Wild population	8	119.45°	27.22°	556
Qixi, Kaihua	Population of ancient trees	9	118.22°	29.23°	371
Huabu, Kaihua	Wild population	8	118.16°	29.01°	152
Qingyuan	Wild population	7	118.55°	27.44°	366
Jiangshan	Wild population	11	118.39°	28.50°	173
Wuyuan	Population of ancient trees	16	117.50°	29.12°	78

The electronic version of this article in Portable Document Format (PDF) will represent a published work according to the International Code of Nomenclature for algae, fungi, and plants (ICN), and hence the new names contained in the electronic version are effectively published under that Code from the electronic edition alone. In addition, new names contained in this work which have been issued with identifiers by IPNI will eventually be made available to the Global Names Index. The IPNI LSIDs can be resolved and the associated information viewed through any standard web browser by appending the LSID contained in this publication to the prefix “http://ipni.org/”. The online version of this work is archived and available from the following digital repositories: PeerJ, PubMed Central, and CLOCKSS.

## Results and Discussion

### Sequencing and assembly results

**Figure 1:**
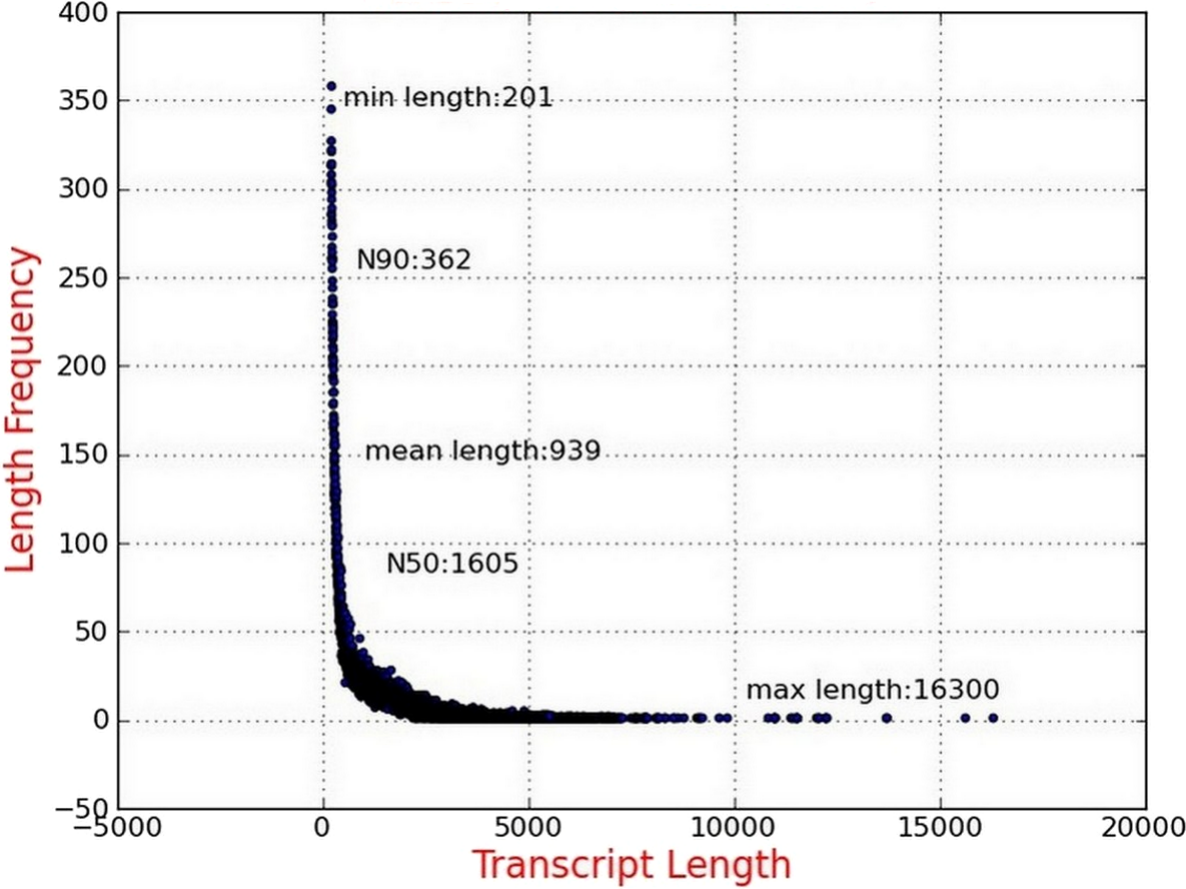
Length distribution of assembled transcripts.

Illumina sequencing data from *P. chekiangensis* were deposited in NCBI SRA database under accession number SRP100128. Two samples were collected and sequenced, and more than 134 million raw reads were initially obtained (Hansen, Brenner & Dudoit, 2010). After the filtering procedure, 128,237,694 clean reads with 93.99% and 93.47% Q30 bases, respectively, were selected for further analyses (Table 2). Using Trinity software, 75,647 transcripts were assembled successfully with an average length of 939 bp and the N50 was 1,605 bp. More than 39,000 transcripts were longer than 500 bp, accounting for 52.81% (Fig. 1). In total, 48,011 unigenes were identified, having an average length of 761 bp, and 19,439 of them were longer than 500 bp (40.49%).

**Table 2:** Summary of *P. chekiangensis* base quality.

Sample	Raw reads	Clean reads	Clean bases	Error (%)	Q20 (%)	Q30 (%)	GC
NM_1	67,268,601	64,118,847	6.41G	0.03	98.30	93.99	47.80
NM_2	67,268,601	64,118,847	6.41G	0.03	97.99	93.47	47.87

### Functional annotation of *P. chekiangensis* unigenes

After the functional annotation (Table 3), 29,714 of the 48,011 unigenes were successfully annotated in at least one database (61.88%) based on NCBI non-redundant protein sequences (File S1), NCBI nucleotide sequences, Protein family, Clusters of Orthologous Groups of proteins, Gene Ontology (GO), the KEGG Ortholog and Swiss-Prot databases, and 3,952 unigenes were annotated in all of the databases (8.23%). Besides, expression levels of unigenes were estimated based on Reads Per Kilobases per Millionreads using RSEM (File S2).

**Table 3:** Summary for the annotation of *P. chekiangensis* unigenes.

	Number of unigenes	Percentage (%)	Functional categories
Annotated in NR	26,693	55.59	
Annotated in NT	10,641	22.16	
Annotated in KEGG	9,132	19.02	31
Annotated in SwissProt	19,828	41.29	
Annotated in PFAM	20,268	42.21	
Annotated in GO	21,164	44.08	51
Annotated in KOG	12,799	26.65	26
Annotated in all databases	3,952	8.23	
Annotated in at least one database	29,714	61.88	
Total unigenes	48,011		

**Figure 2:**
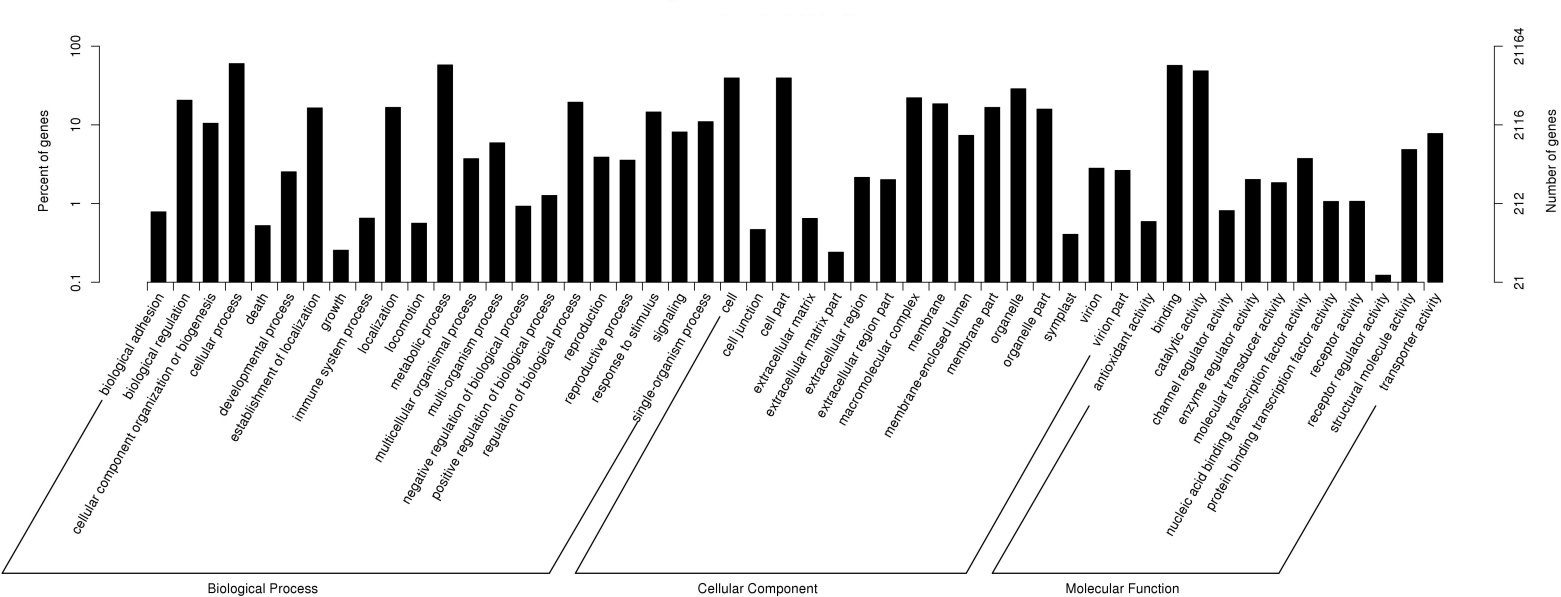
Functional gene ontology classification of *P. chekiangensis* unigenes.

According to GO annotations, 21,164 annotated unigenes were divided into three categories: Biological Process, Cellular Component and Molecular Function (Fig. 2). Then these categories were sub-divided into 51 groups. Out of the 13 second-level groups in the Molecular Function category, ‘binding’ (56.99%), ‘catalytic activity’ (48.54%) and ‘transporter activity’ (7.76%) were the most abundant terms; Out of the 16 second-level groups in the Cellular Component category, ‘cell’ (39.45%), ‘cell part’ (39.44%) and ‘organelle’ (28.75%) had the highest number of unigenes; Out of the 22 second-level groups in the Biological Process category, ‘cellular process’ (60.07%), ‘metabolic process’ (57.79%) and ‘biological regulation’ (20.56%) were the most abundant terms.

**Figure 3:**
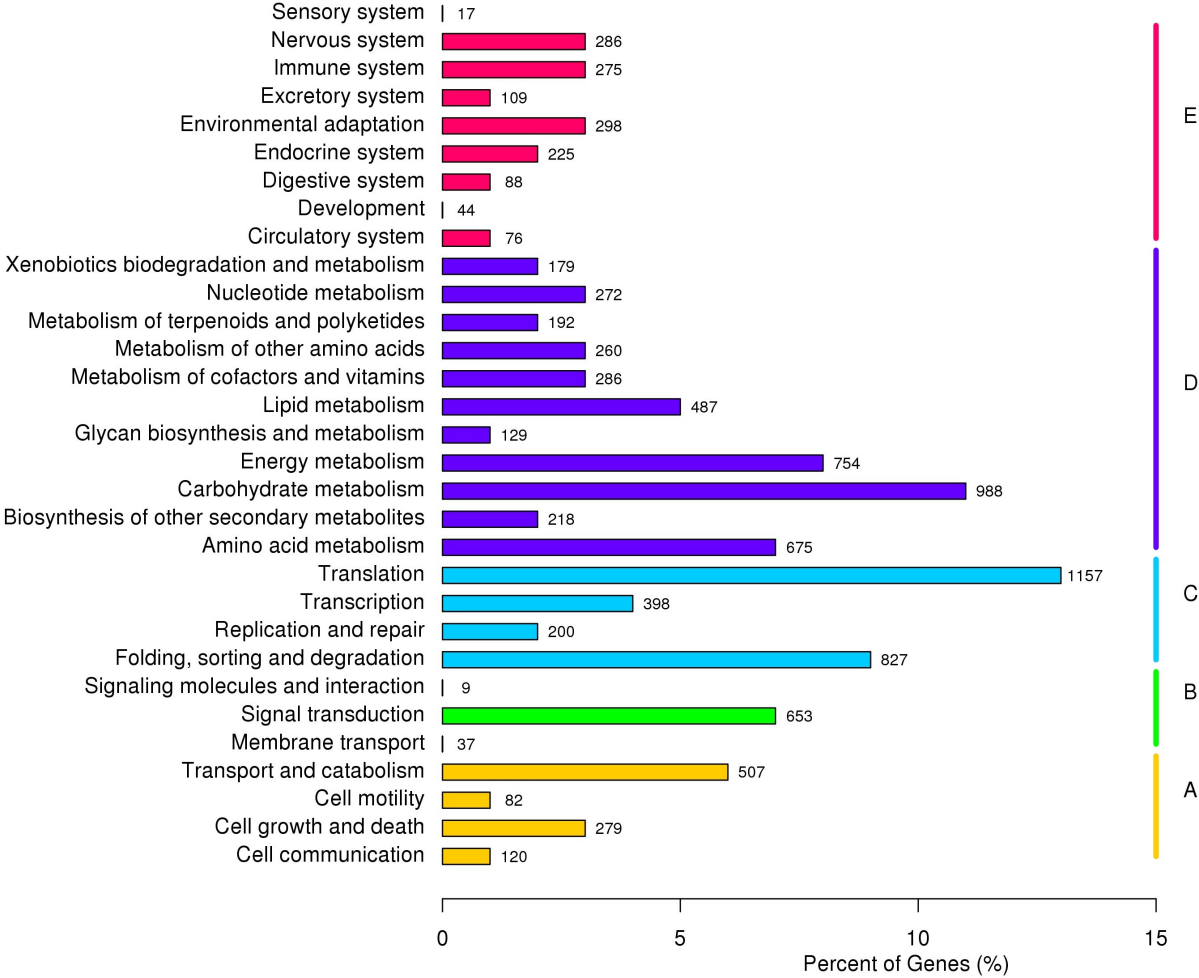
KEGG annotation of *P. chekiangensis* unigenes. All of the unigenes were divided into five subgroups: (A) Cellular processes; (B) Environmental information processing; (C) Genetic information processing; (D) Metabolism; (E) Organismal systems.

Based on KOG classification results, 12,799 unigenes were divided into 26 categories and three richest categories were ‘general functional prediction only’ (15.43%), ‘post-translational modification’ (13.24%) and ‘signal transduction’ (9.90%). According to KEGG annotation results, 9,132 unigenes were divided into five major clades, including 31 sub-terms. ‘Genetic information processing translation’ (12.67%), ‘carbohydrate metabolism (10.82%) and ‘folding, sorting and degradation’ (9.06%) were the three richest sub-terms (Fig. 3).

### Predictions of SSRs and SNPs

A total of 48,011 transcripts were examined for SSR prediction (File S3), and 9,505 were identified with SSRs (19.80%) and 1,830 sequences were found to have more than one SSR. According to the prediction results, 11,776 SSRs were predicted and one unit repeats accounted for the highest percentage (49.03%). In addition, 736 SSRs were present in compound formations.

**Figure 4:**
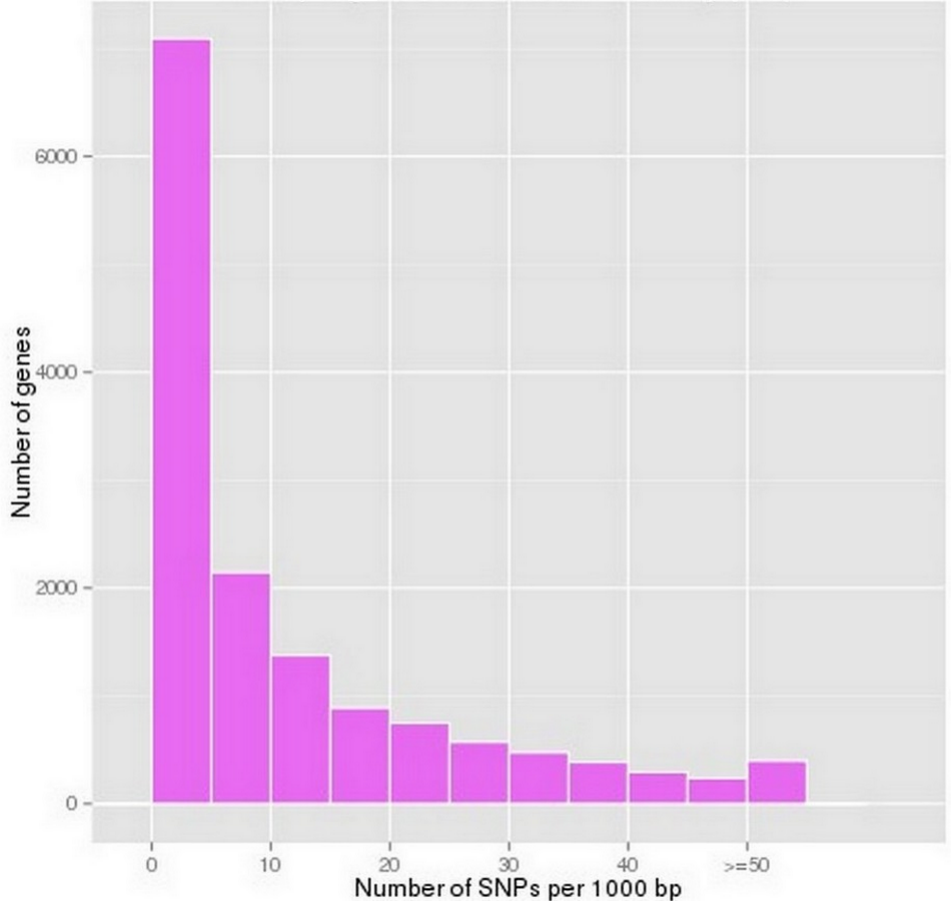
Frequency distribution of SNP densityin *P. chekiangensis* according to predicted results using SOAPsnp.

According to the SOAPsnp prediction results, 162,983 putative SNPs were predicted in *P. chekiangensis* (File S4). Among them, 77.27% were in non-coding regions, 22.73% were in coding regions, 22.60% were synonymous SNPs, and 0.13% were non-synonymous. Most unigenes have less than 10 SNPs per 1,000 bp, indicating that the SNP frequency in *P. chekiangensis* was relatively low (Fig. 4). Although most SNPs seem not to affect the amino acid composition, they may be closely correlated with a bias in codon usage.

### Validation of SNP prediction results

To further validate the putative SNPs, 15 unigenes containing 100 putative SNP loci were selected and primers were designed (Table 4). Because of the limited samples, the putative SNPs were validated using Sanger sequencing results, with the sequences amplified by PCR. All amplified sequences were sequenced accordingly, and when the double peak phenomenon was observed at one locus, based on the sequence diagram together with the comparison results between other sequences, this site could then be defined as a SNP according to the theory of polymorphism. As a result, 25 putative SNPs were finally validated.

**Table 4:** Primers and variants of 25 feasible SNPs in *P. chekiangensis*.

Unigene	SNP locus	PCR primers (F and R)	Allele	Unigene function
comp100159_c0	325	F: GAGGAAAGAAGCTTATGG	T/C	Unknown
		R: TGCATGCGACTAACAACT		
comp100433_c0	567	F: TCAGAATTGCTGACTTGT	G/A	DNA-directed RNA polymerase subunit beta
		R: CATCAATACCAATTGCCAA		
comp102740_c0	416	F: AGTAGTGTGGATCCAACCC	C/A	Unknown
		R: ACTATCTCTATGCATATCA		
comp39317_c0	244	F: TCTAAAATGATGAAAACGA	A/C	Pentatricopeptide repeat-containing protein
		R: AGCAGTTTGAATACATGG		
comp42809_c0	57;103	F: CAAGACAAATCTTGGATT	G/A;G/C	Thiopurine S-methyltransferase
		R: GAAACGGAGATTGAAGTTT		
comp44031_c0	891	F: TGTTAACTCTAATGGCATC	A/G	Lysine histidine transporter-like 7
		R: AAGCATCAGAGAGTGGAG		
comp45316_c1	930	F: GCCGTTCCCTCGAGCCTTG	A/C	myb proto-oncogene protein
		R: GAAGAAGATGAGGGTGGG		
comp41583_c0	523	F: TCCTGCTAATTGTTGAGAC	C/G	Peptidyl-prolyl cis-trans isomerase NIMA-interacting 4
		R: TCATAGGTTATCCATAAT		
comp44876_c0	494	F: CTGCAGAGAAGAAGGAGAG	C/T	Jasmonate ZIM domain-containing protein
		R: AATGTGATAAGAGCCTTTC		
comp44881_c0	153	F: GGGTGAGATCTGAAAAGAAA	A/G	Unknown
		R: GACCGTTGAATTGAAAGG		
comp45780_c0	54;209	F: CATGCGTTTGAAAGGAAGC	A/G;G/T	RNA 3′ terminal phosphate cyclase
		R: GTTAGGATGATTGTCATG		
comp47295_c0	846	F: TCCACCTTACAAGATTTA	C/G	Putative glutamine amidotransferase YLR126C-like
		R: TACGAAGGCTTCGTCATCA		
comp48234_c0	664	F: TTCATCATCTGTCGTCGAA	C/T	30S ribosomal protein 2
		R: CTCGGATGCTCAAGAGAAA		
comp48580_c0	331	F: GTTAAAATGAATTGTTTTT	A/G	Unnamed protein product
		R: AATGTGTCAAGAATACTAC		
comp50565_c0	295;324	F: CGCATGGCGTACAGCCCTA	C/A;A/T	Nucleotide binding protein, putative
		R: TTGAGCAGAAGCTTGACCT		
comp50815_c0	148;252	F: CGGAGGCTCTCGCGGTCTC	T/G;G/C	Putative lipase ROG1-like
		R: ACAAAGACAGAAGGCCAG		
comp531362_c0	256;402	F: CCAAGACTTAAGAAGGGG	T/G;G/A	UPF0481 protein At3g47200-like isoform 1
		R: TATCCACCTCCCTATACAG		
comp5334_c0	363	F: CACGATCGGGCCGAGGAC	C/G	Unknown
		R: TGCCGGTGCGGCACGAGCT		
comp5410_c0	586	F: GCAGCTTCTTCTTCTTCT	C/A	Surfeit locus protein
		R: GATCCAGTGATGAATTGG		
comp544568_c0	257	F: TCTACTGGAGAGGCCAAC	A/T	Pre-mRNA-splicing factor ATP-dependent RNA helicase
		R: TCTTCAGGAGCTCTCTGTT		

### Assembly and annotation of *P. chekiangensis* unigenes

**Figure 5:**
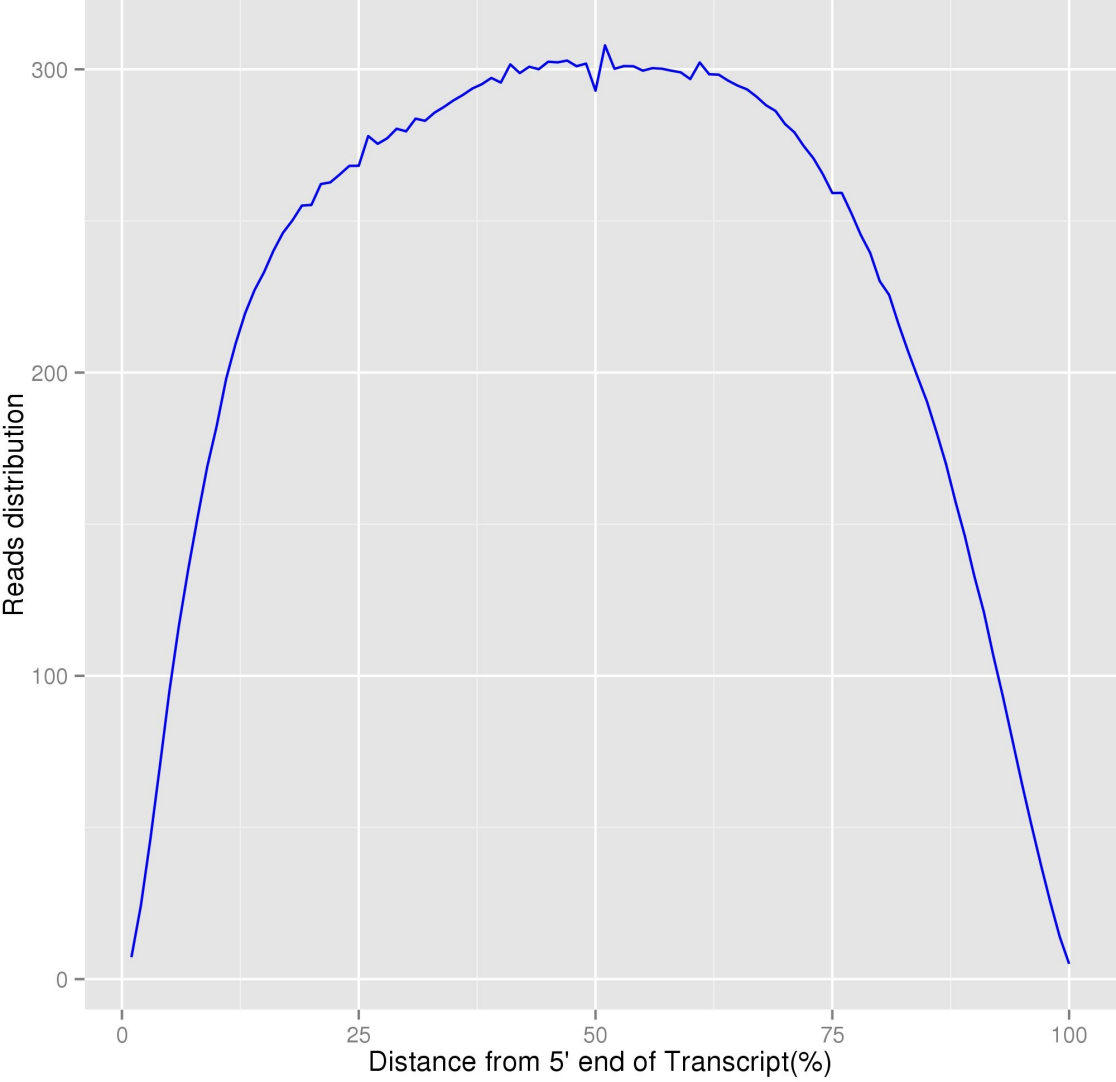
Homogenization distribution curve of *P. chekiangensis* transcripts. The vertical axis represents the average values of sequencing depth.

In our study, 48,011 unigenes were assembled and 29,714 unigenes were successfully annotated. Based on the distribution of homogenization results, although the sequencing depth of the 5′∕3′ regions were relatively lower, the overall degree of transcripts’ homogenization was high, indicating that our transcriptome sequencing results could satisfy the following analyses (Fig. 5). Because limited previous studies have been reported on the molecular mechanisms of this species (Gao et al., 2016), we believe that the assembly and annotation of these unigenes, including eight unigenes involved in lignin synthesis, would be beneficial to the research on *P. chekiangensis* molecular mechanisms, including the exploration of its excellent wood properties. Based on our annotation results, only 61.88% of all unigenes were successfully annotated in at least one database, suggesting that nearly 40% of the unigenes were uniquely distributed in *P. chekiangensis*. Therefore, the large number of unigenes, together with the transcripts, could effectively increase the transcriptomic and genomic information available for this species. Additionally, the prediction of 162,983 putative SNPs and 25 validated SNPs in *P. chekiangensis* may be useful in detailed population genetic analyses.

**Figure 6:**
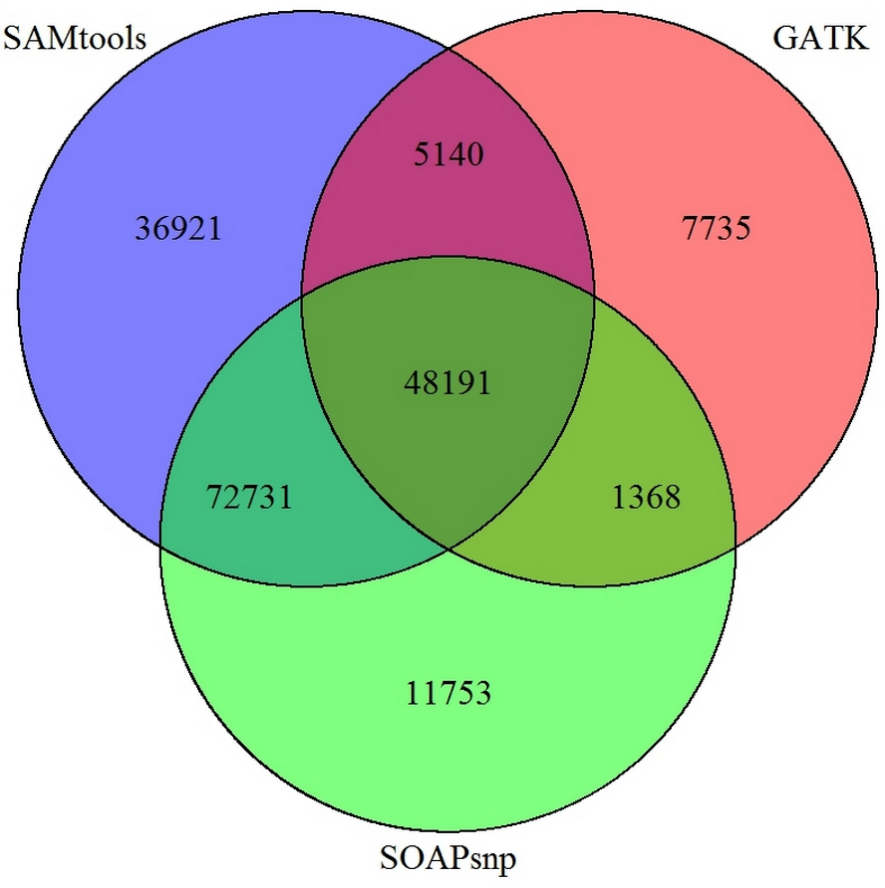
Comparison of SNP prediction results when using three different SNP calling packages.

### The criteria of SNP validation in *P. chekiangensis*

With the appearance of the next-generation sequencing technology, this will allow for the sequencing of polymorphic genotypes on specific target areas and consequent SNP identification, and the direct sequencing of DNA segments amplified by PCR from several individuals is still one classic method to identify SNPs (Oeveren & Janssen, 2009; Portis et al., 2013; Rafalski, 2002). Since we used Sanger method to validate putative SNP results, we sequenced each single sequence from both two ends (5′ end and 3′ end), and then they were assembled together in order to make sure the reported variants were not in fact the product of sequencing. However, in some scarce species with high heterozygosity, such as *P. chekiangensis*, a few problems may not be ignored. One important issue is that when amplified segments were paired-end sequenced, the results may not be easily assembled, and false positive SNPs may easily be detected because of the interference of heterozygosity or misalignment of paralogs. As a result, single sequences in one individual may have changeable bases at one position, and this may be confusing when analyzing multi-sequence alignment results to validate SNPs. As a considerable fraction of the predicted ‘SNPs’ are nucleotide polymorphisms between orthologous regions in the parental haplotype of a heterozygous individual, hence the exact rate of false positive or accuracy may be difficult to determine because of probable alignment artifacts caused by misalignment. In our study, we excluded this situation in one individual and only regarded base differences derived from multi-sequences as validated SNPs Besides , only obvious base differences together with double peak phenomenon observed were regarded as validated SNPs, although the clear definition of polymorphism in a single individual remains a question.

### The results varied when using different SNP prediction software

In our study, SOAPsnp (Li et al., 2009b) was selected as our SNP prediction software. However, more than 20 software packages or programs, including GATK (several versions included) (McKenna et al., 2010), SAMtools (Li et al., 2009a), SOAPsnp have been developed to predict SNP, for both transcriptomes and genomes, regardless of the *de novo* assembly or a reference. In addition to using SOAPsnp for *P. chekiangensis* SNP prediction in this research, GATK and SAMtools were also selected for comparison. The different SNP prediction software packages varied greatly in time consumption and accordance, with an average accordance between different SNP software of less than 25%, indicating that most SNP prediction results were not consistent when using different prediction software (Fig. 6).

Although most SNPs for experimental validation were randomly selected, 53 of them (53%) were common in all three SNP callers. Besides, the number of those SNPs with very high/low quality values was restricted, and unigenes with more accurate annotation results were preferred. However, according to our validation results, only 25% of the prediction results were successfully validated in *P. chekiangensis* using SOAPsnp. Among all three SNP callers, SAMtools seemed to performed best with highest accuracy among the common 53 SNPs (19/53). Considering the limitation of samples, it might be a bit arbitrary to draw the conclusion that SAMtools is better than other two SNP callers. However, it should be noted that an even greater proportion than 75% (75/100) are false positives using SOAPsnp, although some of the variants reported may be real, the vast majority should be expected to be false. Thus, there should be numerous Type I or Type II errors in predicting SNPs when using different software. Determining which software is more suitable for various kinds of datasets (based on accuracy and precision) would be an interesting issue for further work.

##  Supplemental Information

10.7717/peerj.3193/supp-1 File S1 Annotation of *P. chekiangensis* unigenes Annotation information of assembled unigenes according to Nr, KEGG and other databases in *P. chekiangensis.*

10.7717/peerj.3193/supp-2 File S2 Expression level estimation of unigenes Using Reads Per Kilobases per Millionreads (RPKM), the expression levels of unigenes were estimated in *P. chekiangensis.*

10.7717/peerj.3193/supp-3 File S3 Predicted SSRs in *P. chekiangensis* The results of putative SSRs using Misa.

10.7717/peerj.3193/supp-4 File S4 Predicted SNPs in *P. chekiangensis* Putative SNP results and their loci according to SOAPsnp in *P. chekiangensis.*

10.7717/peerj.3193/supp-5 File S5 Perl script for data trimming Perl script for quality check and raw data trimming of fastq sequences.

## Additional Information and Declarations

### Data Availability

The following information was supplied regarding data availability: The raw data has been supplied as a Supplementary File.

### New Species Registration

The following information was supplied regarding the registration of a newly described species: 77067542-1—*Phoebe chekiangensis*.
